# Analectic

**Published:** 1833

**Authors:** 


### ANALECTIC, ANALYTICAL, AND ORIGINAL.

### I. Analectic.

*Ad Sylvas Nuncius.*

1. *A Sketch of Broussais' Doctrines, drawn up by himself.*—It was in the year 1804 that I began to be sensible of the imperfect and erroneous notions of disease, which were then prevalent. I labored diligently for five years, and in the year 1809, appeared the "History of the Chronic Phlegmasiæ." At that time I was at a distance from Paris, and engaged in the army hospitals. The work was one of an entirely experimental nature, and obtained an honorable mention from those who were able to judge of its merits. At the time at which it was published, the attention of physicians had not been at all directed to the subject of chronic inflammations, especially of those which have their seat in the membranes lining the visceral cavities. Pinel does not even mention them in his Nosographie. In their place we find the unmeaning terms "organic diseases, or defects, and atrophy or decay without any appreciable cause." Corvisart too was ignorant of the true nature of internal morbid changes. If in a case, which was neither consumption, nor disease of the heart, nor any of those inward swellings denominated at that time organic alterations, he observed the progressive decay of a patient affected with a chronic malady, he attributed it to a state of mere feebleness and cachexy, (malus habitus;) expressions vague and utterly unmeaning, or rather we should say extremely mischievous, as leading to erroneous indications of treatment. The History of the Chronic Phlegmasiæ corrected these faults, and shewed that inflammation is the chief agent in the production of those diseased growths and changes which we meet with in the centre of the viscera, and also in the texture of their lining membranes, and that these are the real causes of that incurable exhaustion and decay, which had hitherto been always referred only to debility of the solids, and depravation of the fluids of the body. Moreover it proved that such debility and such depravation are often curable; it pointed out the signs and epochs of their curability, and also the means for their cure. Henceforth the term "organic disease"

had a definite and appreciable meaning; medical men were satisfied in attempting to soothe, and not vainly to eradicate such; and their attention was chiefly directed to prevent its occurrence, or progress, as soon as they discovered the germ in those obstinate and intractable irritations which equally invade all structures and tissues. The treatment of such cases was no longer empirical, but rational and consistent. The class of fevers was at this period as imperfectly understood, as those of cachexies and of organic diseases. Continued fevers were divided into two sorts; the one attributable to the inflammation of a particular organ; the other being viewed as essential, or independent of any local affection: but such a phrase as "essential" means nothing; for we are still puzzled to say, to what cause are we to ascribe an essential fever; in this state of ignorance, the physicians tried to characterize such a case either by the predominating symptoms, or by vague and idle conjectures and speculations; if the secretion of the bile was much increased, the fever was termed "bilious;" if that of the mucous surface in any part, a "mucous or pituitous" fever; when the temperature of the body rose high, then it was a "burning" fever; and when it fell much, it was a "cold, or algid" fever; again fevers were called asthenic, putrid, nervous, and ataxic, as they happened to be attended with exhaustion, with putrescency, and with symptoms of disturbance of the nervous and muscular symptoms; there were also hospital, camp, and prison fevers: and fever with petechial, miliary, or other eruptions. In some cases, the character and name were drawn, it would seem, from an idea that a baneful agent was working its effects upon the patient, and defeating the physician's efforts; and hence such fevers were styled "malignant." Does this language convey any knowledge? or does it not rather prove the confusion and chaos in which the subject was involved, and mislead the practitioner not only in his attempts to reason on the nature of the diseases, but also in his efforts to cure them? Hence one set of men was drawing the indications of their treatment from the state of the biliary, or nervous secretions; while another set was talking only of debility, putridity, or malignancy. Pinel was the first to perceive, and to aim at correcting these errors of the schools. He tried to localise some fevers, as for example the bilious fevers, under the name of meningo-gastric, and the pituitous, or mucous fevers, under that of adeno-meningeal. But he has not told us in his Nosographie, of what nature is the gastric disorder, nor yet of the causes of the increased quantities of mucous, which is characteristic of the second class; moreover he fell into the egregious contradiction of attributing these two sorts of fevers, sometimes to irritation, and sometimes to a primitive change of the biliary and mucous secretions, at the very time that he designated them, essential fevers. Then he vaguely ascribed all his ataxic fevers to a disorder of the nervous system, and most confusedly classed together all fevers in which the vital energies are depressed, and termed them adynamic. Not only was there no localisation of the disease, but the indications of treatment in such a system were most injurious: for what is the cause of the loss of power, or debility in fevers, but simply inflammation of some of the viscera? Such was the state of the science, when I published, in 1816, the first edition of my Examination of Medical Doctrines. In it I pointed out the absurdity of expecting to advance the science of medicine, merely by forming groupes of symptoms into particular diseases, which is not less absurd than to say that an assemblage of ten or twelve symptoms is the virtual cause of those morbid changes which we observe after death. The symptoms in short are nothing but the external and obvious evidences of the malady, which is involving the organs in abnormal changes. I proposed to view fevers, as we do inflammations, to localise them in certain parts, or organs, and I attempted to show that the morbid changes of structure which are going on, are the real existing causes of fevers, and not, as had hitherto been supposed, the effects of them. At the same time, I strongly inculcated the propriety of extreme caution in our reasoning on those cases, where the "mobile" of the fever is not apparent. These new doctrines encountered much opposition, but they gradually gained ground, and became more and more adopted, as will be seen by the following events. In 1812, was commenced the large "Dictionnaire des Sciences Medicales," the articles of which were deeply imbued with the doctrines of Pinel, till the year 1817, from which time some sprinkling of the physiological reasonings began to appear. Before the work was completed, the "Dictionnaire Abrege" was brought out, and in it the physiological medium was preponderant. In the large dictionary, fevers were enumerated as "essential, or idiopathic;" in the abridged one, we read of none but such as are symptomatic. In both, the doctrines which I had unfolded in my History of the Phlegmasiæ, were insisted upon, in discoursing upon all the diseases which are included in the catalogue of inflammations. For the last sixteen years, almost all medical researches have taken their rise from, and have been based upon the physiological doctrines. The subject of inflammation is studied, discussed, and specialized, much more than it was formerly; and where inflammation cannot be proved to exist, irritation is supposed to be present, and is now believed to be the agent in producing a host of organic diseases, which were before merely enumerated in detail. The relations between the changes and vitiations of the fluids, and the different sorts and degrees of inflammatory and irritative affections, are attentively and most zealously examined. In 1821, appeared the second edition of my "Examen des Doctrines" which was published originally in one volume, then in two, and at length in the fourth edition in four volumes. Soon after this period I began my system of "Physiology applied to pathology;" it was brought out in numbers for four successive years. It is devoted to the examination of the etiology of diseases, and to the tracing of those functional aberrations from the healthy to the diseased state, which are caused by the influence of the external agencies continually operating on the living body. This work, as well as the preceding ones, has received the honor of translation into several languages. In addition to these, the "Annales de la Medecine Physiologique" have appeared in monthly numbers for the last eleven years, and my Treaties on Irritation and Madness in 1825, in which last work the cause of the "positive philosophy" was pleaded with a boldness and a freedom, which might have been dangerous to the author. Lastly the cholera morbus, which has so puzzled and bewildered all medical enquiries, must be viewed through the enlightening medium of correct physiology, before we can hope to arrive at a successful therapeutic. We have explained our views upon this subject in an essay which was lately published. According to the physiological school of medicine, no disease is ever primarily and essentially general, that is, affecting equally at once every part of the system. Disease begins always in one individual organ, even when the disease depends upon a cause which induces an alteration in the humors, or fluids of the body, such as the exciting cause of small pox, typhus, &c. &c. The great aim of the practitioner ought therefore to be, to discover the primitive seat of the disorder, as he may thereby often be enabled to check, and even strangle the nascent evil. By following this mode of examination, French authors have surprisingly reduced the catalogue of malignant fevers, which are now seldom met with, except when medical assistance has been called too late, or has not been accepted. Look at the reports of the different hospitals, and mark how seldom we find the term of general or essential fevers, and how prevailing is the belief that all diseases ought to be regarded primarily as local affections. Even when the physician does not see the case, until the disturbance has been propagated to, and has involved all the functions of the economy, he can do much to relieve his patient by following the physiological method, and directing his attention to those organs which have chiefly suffered. Hence we perceive the absurdity of recommending in fevers only one set of remedies to the exclusion of all others; the remedy must be adapted to the condition and susceptibility of the affected organs. The operation of all agents which influence and modify the state of the system, ought to be our especial study, and the effects of these upon the moving and sentient powers, our only guide in judging of the results. Thus we admit no a priori reasonings and conclusions, into our school; neither any preconceived opinions, nor any implicit following the "verba ullius magistri." Let it not be supposed that our doctrine is that the abnormal changes and modifications, which we have alluded to, are the only immediate cause of all diseases; we know that these causes may exist in the humors, in certain poisons, in the vicissitudes of heat and cold, in the imponderable agents, and probably also in influences which cannot be appropriated by our senses, and we are not opposed to any researches calculated to explain or illustrate the action of these causes, and to discover antidotes to them, but we simply maintain that a disease can be recognized only by the aberration from the normal condition of the moving and sentient powers of the body, and that the study of this aberration, sometimes as excessive, at other times as impaired, or irregular action, can alone furnish to the medical man the correct method of ascertaining whether his remedies are beneficial, or injurious. Formerly a patient, who was getting worse, instead of better, while under medical treatment, was often told, "Have patience, it is the medicine which is acting upon you;" a gouty man was consoled by hearing that his physician must not relieve him, for his sufferings were in sooth necessary for the object which nature had in view; and even in many acute diseases, the patient was congratulated on any increase of fever and restlessness, upon the ground that a salutary crisis of his malady was at hand! How often was the raging thirst of fever met with hot drinks, which only add fuel to the distress! We have a memorable instance of this folly in the late treatment of the cholera; but now, thank God, the poor sufferers are permitted to have as much cold water and ice, as they desire. There are indeed cases, in which the patient must submit to the annoying effects of certain medicines: but such cases are infinitely more rare than was formerly believed; but yet we must acknowledge that much injury is often done by their employment; thus, in dyspepsia, how frequently do we see stimulants and tonics ordered, although it is obvious that they must either increase the distress, or only change its character. In short, the art of sparing suffering and pain is not so ancient as many suppose, and has been fostered chiefly under the happy influence of the physiological method; for the very essence of this method is to bring relief to suffering organs, and not to regard diseases merely as assemblages of certain symptoms, which must either co-exist, or succeed to each other; by this foolish practice we neglect the very increase of those evils which are successively added to the inseparable distresses of the malady. Is it not a common remark of a disciple of the old school, when summoned to a patient—"we must wait; the disease has not yet declared itself?" But such dangerous absurdities are gradually passing away; the mind of the physiological physician is occupied with facts and realities, and not with preconceived and a priori reasonings.

In enquiring into the nature of a disease, there are but four objects of research—1, The organ primarily affected. 2, The operating cause by which it has been affected. 3, The influence of this organ upon the other organs of the body; and, 4, The agents by which the disorder may be removed. There is nothing of idle speculation here—no empty hypothesis on the intimate or proximate nature of diseases, for these are beyond our intelligence; we do not pretend to give an explanation of the phenomena of life; our only aim is to take notice of whatever disturbs its moving and sentient powers, or disorders the texture of the solids and the composition of the fluids, and to compare it with whatever has the effect of restoring the one and the other to a state of normal rectitude. How, then, can any one be so unjust as to denominate the physiological method, a system of a priori reasoning and practice, or as a monomania, or a partial, contracted, and exclusive doctrine, which attends only to one set of measures, and disdainfully rejects every other?—*Annal. de Med. Physiol. June,* 1832.

In order to unfold the views of this distinguished reformer of medical science, we are induced to give a short abstract of the essay which he read in October, before the Academy of Sciences of the Institute at Paris, as a candidate for the chair vacant by the death of M. Portal. It is entitled "Memoir on the Philosophy of Medicine."

He remarks at the outset, that, at different epochs of history, very different opinions have been held on the meaning of the phrase "medical philosophy." Hippocrates deemed it to consist in closely observing the march of diseases, when they are little modified by the interference of art. Galen made it to reside in the study of the humours; Paracelsus and his astrological followers, in that of the stars; Vanhelmont and Stahl brought it down again to earth, and fixed it in the centre of every organ, designating it the "archæus" and the "animus" which presides over the body. At length, the immortal Haller shewed that it was more philosophical to search for its abode in the irritability of the tissues, and to ascribe health and disease to certain states of these, rather than to subtile and irritable nonentities. But soon a new set of theorists arose, who maintained that all the phenomena of life, both in health and disease, are referable to the action of the nerves; but, unfortunately, the nervous functions are little known, and in course of time a being or essence, semi-intellectual and semi-material, partaking somewhat, too, of the nature of the "archæus" of Vanhelmont, and of the "animus" of Stahl, was admitted into all medical reasonings, as the presiding influence of the animal economy. This new philosophy was simple, to all appearance; but it was speedily split, and two rival symptoms sprung from the division. In the one, which was upheld by Barther and the Montpellier school, a vital force or energy was made to reign over all the tissues and humours of the body; but its seat was not limited to the nerves, as was the case with its parent doctrine; according to this philosophy, there were as many vital forces as there were vital phenomena: in the other, the elements were "force" and "debility," terms which are synonymous with an "excess and deficiency of" excitability, or with "accumulated and exhausted irritability." Such was the Brownonian doctrine, a doctrine which led to a most pernicious system of therapeutics, for remedies were now employed with such whimsical and irrational intentions, such as to increase or diminish excitement, to affect the irritability, as it chanced to be redundant or defective, just as, before, they were addressed to the archæi and animi, and with equal disregard to the effects which they produced on individual organs. It was Sydenham who first proposed the classifying of diseases; Sauvages seized the happy idea, and, comparing diseases (which are only modified conditions of a living body) to plants (which are living bodies themselves,) gave birth to nosology! Nothing was talked of but classes, orders, genera, and species, as in botany; his adage was, "symptomata se habent ad morbos, ut folia, et fulcra ad plantas," an adage whose absurdity must now be apparent to every one, but whose influence, strange to say, has not yet entirely passed away. It is surprising that no one could see the folly of comparing pain and heat to a leaf and a branch, and a spasm to a flower!! It is useless to advert any longer to these idle abstractions and vagaries of the brain, or to expose more of the ridiculous conceptions which other authors have, at different times, propounded; for surely medical "ontology" is for ever abolished. It was formerly the great problem among physicians—"a disease being given, to find its remedy;" but latterly the problem had been somewhat changed, or rather a new problem was in vogue—"a disease being given, to find its place in a nosological catalogue." How humiliating to think that the minds of men were so diverted from the true path of enquiry, and that they allowed their judgments to be blinded and misled by the ridiculous nothingness of empty, sounding words; but let us not boast, but rather ponder on these errors, for the improvement of ourselves.

Our next theme is, to strive to find out some more rational system of medical philosophy. The eclectic school have put forth their claims; they tell us "there is something good and true in all the preceding doctrines—let us only reject the bad and retain the good." Such is the method of those who profess not to be "exclusives." But we need not waste time in refuting the reasonings of the eclectics, a name which ought to be reserved for close observers and able experimenters, whose time and talents are engaged in verifying facts which have been long admitted, and in seeking after others which are novel and interesting. How is it possible for an eclectic to arrive at any logical correctness, if, surrounded with so many statements and data, brought forward by one party and refuted by another, and encountering so many opinions and hypotheses utterly conflictant with each other? What could a "neophyte" do amid such a labyrinth of perplexities? Eclectics are not known in the true sciences, where the simplicity and the small number of facts appertaining to any theorem, render its demonstration easy and intelligible. The great error of most preceding writers has been, in seeking after the primary and proximate causes of disease; and, even in the present day, we hear men often regret the misfortune of not being able to attain to the knowledge of the essential nature of diseases; they are unwilling to admit any evidences or elements of certainty and truth in science, but such as are proved by the testimony of the senses, forgetting that there is another kind of evidences obtained, by inductive reasonings, from facts which have been well established; and such evidences are not less real than a shadow, a noise, a reflection, or evidences of a material object existing somewhere; they are admitted into mathematics, chemistry, mechanics, agriculture, and exercise a mighty influence in all discourses upon these sciences.

None can be ignorant of the stupendous results derived, through this means, by Cuvier in fossil geology. It is, indeed, difficult to obtain this sort of evidence in medical researches, but the cause of this is, that the facts, or rather data, are so very numerous and complex; but we must not be discouraged, for it is only by the medium of such evidence, that we can hope to arrive at a knowledge of the causes of disease—to a correct nosology, and a rational and beneficial system of therapeutics. This class of evidences is weak and deficient at present, but we must labour zealously in enriching its domain, for it constitutes the most important aim of medicine; yet such is the medical philosophy of our days, that rigid induction has been less esteemed than mere description; the former is falsely designated as an hypothesis, theory, a system a priori, and an empty conjecture. If a treatise on any disease, the only aim and end of the author are, after describing its course and progress, and detailing the pathological changes in fatal cases, to establish that the disease could not be at all different from what it really was, or that the symptoms were merely the effects of the morbid states and actions which were going on in different viscera, and no attention is paid to appreciate the operation of the exciting cause, and of the agencies which modify the actual state of disease, and scarce any thing is thought of but the hunting after nostrums and specifics. The habit of expecting a necessary succession of symptoms during a definite period, in most maladies, and especially in those which have been denominated putrid and malignant fevers, typhus gravior, &c. is most pernicious and baneful. We have labored to expose the dangers of such a method, and we deem it one of the noblest endeavors of a physician to shew, that the greater part of these affections may be arrested at the different epochs of their development, by means of a scientific application of those agents which modify and control the functions of our organs. In conclusion, the philosophy of medicine which we have proposed, consists in a more rigorous observation of the effects of all the external agents on the human frame, and of the influence of the different organs of the body on each other.—*Archiv. Gen. Oct.* 1832.—*Med. Chir. Rev.*

2. *Death of M. Portal.*—This distinguished veteran died on the 23d of July last, at the good old age of 91, as rich in honors as in years. He was the Nestor of the French Physicians; first physician to Louis XVIII. and Charles X., perpetual president of the Academy of Medicine, member of the Academy of Sciences, and of many other celebrated societies. His "History of Anatomy and Surgery" has long been esteemed and admired; but his greatest work was the "System of Medical Anatomy," which laid the foundation of, and rendered popular, the pursuit of sound pathology in France.—*Ib.*

3. *On Water, applied externally, as a Remedial Agent.*—In our last Number, we gave an extended analysis of a very valuable paper on the above subject; we now continue the subject, and shall direct our readers to the fittest method of employing the remedy. In most cases, especially those in which the nervous system is much affected, as in the 3d, 4th, and 5th series, it is of importance that the bath, should be in the bed-room, or in an immediately adjoining apartment as the patient ought to go to bed immediately afterwards; in other cases, as in those of the 1st, 2d, and 5th series, it is better that it be at some distance, as the exercise of going to and coming from the bath is beneficial. The heat of the bath ought to be about 86 degrees, or thereabouts; the affusions ought to be made with the water of the bath at first, and then gradually with cooler water. As a general rule it may be said that the temperature of the bath should be about 86 degrees, and the water for effusion from 6 to 10 degrees lower. With regard to the duration of the bathing, this must vary according to the nature of the malady. In the cases of the 3d and 5th series, those, namely, characterized by lethargy and nervous stupor, from five to eight minutes is long enough; but in cases of the 1st, 2d, 4th, and 6th, series, viz. those of cerebral fatigue and exhaustion, fatigue of the brain and of the organs of sense, nervous fevers, and neuroses of the sympathetic nerve, 25 to 30 minutes may be mentioned as a proper time, the effusions being employed during the six or eight last minutes. Now the number and frequency of the baths is also a point of importance to determine: when the patient is afflicted with stupor, nervous fever, or lethargy (as in the 3d, 4th, and 5th series,) it is seldom that a single bath in the 24 hours is sufficient; generally two baths at least are necessary; sometimes 3, 4, or even 6 and 8, according to M. Recamier, must be taken in one day, before the patient experiences decided benefit, whereas, in instances of the 2d and 6th series, one bath daily is amply sufficient. A third interesting question of inquiry is, the proper hour of the day for the use of the bath. In cases of the 1st and 6th series, the most fit time is shortly before the evening repast, or not long before going to bed; the appetite, in the one case, is often much invigorated, and in the other, sleep may probably be soon induced; it may be proper that the patient should take a light supper after he is in bed—*Revue Medicale, Sept.* 1832.

*Remark.*—We have repeatedly witnessed the most agreeable effects from the employment of bathing, in a somewhat similar form to that recommended by the French physicians, in many cases of cerebral exhaustion, of melancholy, of general debility uncomplicated with organic disease, &c.; we suggest the cotemporaneous effects of effervescing draughts, such as the seidlitz powders, to the more robust, and the citrate of ammonia to the more feeble and nervous. The wonderfully soothing effects of a saline purgative are not very generally known, and may probably be rather despised, by those who have never experienced their effects on themselves, or witnessed them in others. Suffice it to say, that Lord Byron mentions it of himself, that when a fit of the "blue devils" was impending over him, a spoonful or two of Epsom salts always restored his spirits more quickly than the finest wines, and we know a writer, of distinguished talent, who confirms the truth of the remark by his own practice.—*Ib.*

4. *Ulceration of the Stomach.*—Dr. Graves visited an unmarried lady, aged 25 years, who had suffered much from pain in the stomach, nausea, vomiting, acidity, and indigestion. These symptoms first appeared after a severe shock and fright. She had sometimes intervals of five or six weeks at a time, free from complaint, but these intervals had lately become much shorter. She was pale and emaciated. She was free from pain for some months after Dr. Graves commenced attendance, then pain came on attended often with a discharge of colored fluid from the stomach, sometimes resembling coffee-grounds. This state continued for some months, when the pain and tenderness disappeared, though vomiting remained, after every kind of food. She was at length worn out. On dissection, an ulcer was found in the neighborhood of the pylorus, but completely cicatrized. It had penetrated the mucous and muscular coats, but had been arrested by the peritoneal covering, there was no maturation or scirrhus in the neighborhood. The site of the cicatrix was distended into a kind of pouch, which received a portion of the contents of the stomach, and thus formed a kind of valve which prevented the egress of chyme through the pyloric orifice, nutriment was thus cut off from the system, and death was the result.—*Dublin Journ. No. V.—Ib.*

5. *Dr. Graves on Periostitis.*—Dr. G. divides periostitis into two kinds—diffused and circumscribed. The former is that which usually terminates in necrosis, and falls to the care of the surgeon; the latter constitutes nodes. It is to the latter affection that Dr. G. limits himself, in a clinical lecture delivered at the Meath Hospital.

Circumscribed periostitis may arise from cold; most commonly from a specific cause, as syphilis, mercury, or scrofula.

It presents some varieties. First, it exists without detachment of the periosteum from the bone. The membrane becomes inflamed and thickened; the bone beneath vascular. There is now pain and tenderness in the part, sometimes with swelling and discoloration of the integuments. The affection becomes chronic, the symptoms rather decline. Then the periosteum becomes more dense, even fibro-cartilaginous. When this change has taken place, Dr. G. thinks it doubtful whether the diseased mass is ever again absorbed. We are not so sure of this. We see now and then, rarely, we admit, the most solid and bony nodes diminish under the influence of mercury. If healthy bone and fibrous tissue admit of absorption, why may not new osseous or fibrous structure? When ossification takes place in a node, the external lamina of the true bone beneath is in process of time absorbed, and, at the same time, a cancellated structure is developed in the node, the two orders of cancelli thus becoming continuous. The tibia of prostitutes are often much disfigured, from irregular bony elevations of this description.

Secondly, periostitis may be attended with detachment from the bone beneath, and of this there are several varieties. The first variety is this. In a space, varying from twenty-four hours to eight or ten days, an elevation appears on some part of a bone, with pain and tenderness on pressure, forming a tumour apparently solid, but, on accurate examination, somewhat elastic. Soon a gradual diminution of the pain and swelling occur, the fluid effused under the periosteum is absorbed, and the subjacent bone and periosteum become again united. This process generally occupies some time, but occasionally is more speedy. The tumours which rapidly form and disappear on the scalp are of this nature. The surface of the bone does not die. Usually ulceration of the skin does not occur in this variety; but, if it does, the cure is effected by granulations arising from the vascular surface of the bone. In the second variety, matter is effused beneath the periosteum, the bone affected becomes vascular at a little depth, but the surface is white and dead, and consists of a thin, worm-eaten, cribriform lamella, which, after some time, separates, and opens for itself a passage through the integuments. The cure is effected by granulations from the vascular part of the bone beneath.

Scrofulous periostitis is a simultaneous affection of the bone and periosteum. Sometimes, in vitiated constitutions, the periosteum becomes affected, from ulceration commencing in the skin, as in rupia, ecthyma, &c. In all, except the first species of periostitis described,

Dr. G. recommends cutting through the integuments and periosteum, as recommended by Mr Crampton.

The scrofulous periostitis (node) is attended with milder symptoms; less pain and tenderness; less swelling; and occurs at an earlier period of life. Not but scrofula may be combined with the deleterious effects of mercury, or with syphilis. The history and the circumstances must determine this. Mercury and syphilis, too, may combine to produce node, indeed it is probable that, in most cases of node, this is the fact. Some of these cases are most perplexing and most miserable.

Dr. G. adverts to the treatment. As local means, leeches, blisters dressed with savine ointment, the antimonial ointment; in obstinate cases, cutting down to the bone; when ulceration takes place, and there are pale granulations, the introduction of a stick of nitrate of silver, and touching a given part of the surface every day. As to the general treatment, when the constitution is strong, and there is otherwise no objection, the use of mercury; and this Dr. G. considers the best mode, if the symptoms be violent, even though mercury should have been already given. Mercury carried to decided salivation, and continued three or four days afterwards, is particularly suited to the painful species of cranial periostitis already alluded to. When the symptoms are less violent, the pil. plummeri, blue pill in alternative doses; and in debilitated persons, the corrosive sublimate perhaps, or De Velno's vegetable syrup. Dr. G. has seen several restored to health by the agency of the latter. A great objection to the use of mercury among the poor, even in hospitals, is their subsequent inevitable exposure to cold.

Next to mercury, Dr. Graves ranks colchicum and tartar emetic; the former preceded by bleeding, and combined with an opiate and magnesia. Colchicum cannot well be combined with antimonials, on account of the disturbance of the stomach which they occasion. Narcotics are serviceable, during the whole course of the disease. When it becomes chronic, sarsaparilla may be given, with nitric acid.

We think these observations of Dr. Graves not unworthy of attention. The point to which we would particularly direct our readers' notice, is the employment of incisions when the pain is severe, the tension of the periosteum considerable, and inflammatory action present. We are quite alive to the powers of mercury in acute fibrous inflammation, and, indeed, in chronic; yet still, on the whole, we would rather do without it if we could. There is too much reason to suppose, that node would seldom occur as a sequela of the venereal *per se*, and even when, by mercury, we arrest the acute inflammation, we suspect that we are laying the foundation, in many cases, of ulterior mischief; of more extensive inflammation of the bone and periosteum. In the treatment of periostitis, we would rather try strict antiphlogistic treatment and incision first; and, if these failed, we would reluctantly be driven to mercury. It is with node as with some forms of the pustular and tubercular eruption: if we are too easily persuaded to introduce mercury, we not unfrequently complicate the case still farther, and diminish the chances of the unfortunate patient.

*Lond. Med. and Surg. Journ.—Ib.*

6. *Chemical Pathology of the Blood in Cholera.*—By M. de Canu, Professor to the School of Pharmacy at Paris.—The most remarkable and obvious change, is the singular increase of the proportion of the solid matters, to the watery portion; the following experiments clearly demonstrate this fact:

| | | | | |
|---|---|---|---|---|
| In the first: | | | | |
| | Water . . . . . | 66 | | |
| | Solid Matter . . . | 34 | | |
| | | 100 | | |
| In the second: | | | | |
| | Water . . . . . | 74.9 | Mean proportions. | |
| | Solid matters . . | 25.1 | Water . . . | 63 |
| | | 100 | Solid matters . | 37 |
| | | | | 100 |
| In the third: | | | | |
| | Water . . . . . . | 48 | | |
| | Solid matters . . | 52 | | |
| | | 100 | | |

In healthy blood, the proportion of the watery part is about 0.78; and if we take this as a standard, the blood of cholera patients contains sometimes twice the normal quantity of solid materials; hence we can readily explain the treacle-like appearance of this fluid, as it flows from a vein in the arm. I have not detected any considerable changes in the fibrine, albumen, and colouring matter; this last, indeed was of darker hue in the arteries, and seemed to have experienced a sort of modification, analogous probably to that which takes place in the conversion of venous into florid blood. In attempting to ascertain the exact proportions of the different constituents of cholera blood, much uncertainty arises from the difficulty of separating the serous portion; but my observations do not agree with those of Hermann, of Moscow, who states, that cholera blood offers sensible traces of acidity. The relative diminution of the alkaline carbonates is always appreciable, sometimes indeed to such a degree, that they can with difficulty be detected; and since these salts, as well as albumen, and an extractive matter, which has been compared to osmazome, are found in the stools, we may fairly attribute the increased thickness of the blood to the draining of the serous part by the intestines.

The white and apparently fibrinous matter observed in the rice-water stools, seems to me, to be rather of the character of mucus, than of fibrine; and I am more inclined to this opinion, as the fibrinous portion of the blood was not found defective.—*Ib.*

7. *Treatment of Hooping Cough.*—By Professor Autenrieth.—No internal medicine is given, except for contingent symptoms, and the only remedy used is friction with tartar-emetic ointment (ʒiss add ℥j. axungiæ.) A portion of the size of a nut, is to be rubbed on the epigastric region three times a day. On the second or third day, an eruption of vesicles and bullæ appears; the friction is to be continued: the eruption spreads, and becomes pustular like that of small-pox; the friction may now be applied to any other part, but the eruption on the epigastrium is not to be permitted to pass too quickly off. In order to insure the efficacy of the treatment, we must not be satisfied merely with producing pustules; the rubbing ought to be continued till small ulcerations in the intervals between the crusts appear. The above treatment is to be steadily pursued for 8 or 12 days; if the eruption prove very painful, no application is so good as a hemlock fomentation.

Autenrieth has found this method most successful in two severe epidemics, during which he did not lose one patient. He does not approve of the watery solution of the tartarised antimony, and of the tincture of cantharides, recommended by Struve. Henke, an eminent German physician, has seen numerous cases of pertussis much benefitted by a combination of bark and opium; he has also employed, with advantage, various stimulant antispasmodics, as ætherial preparations, ammonia, &c., but very judiciously urges the practitioner to draw the distinction between inflammatory and spasmodic hooping-cough, as it must be quite unnecessary to say, that a plan of treatment applicable to the one form may be most pernicious in the other. —*Ib.*

8. *On the Reproduction of the Crystalline Lens, after the operation for Cataract.*—In the 14th vol. p. 384, of the Philadelphia Journal of the Med. and Phys. Sciences, we gave an account of the experiments of M. M. Cottreau and Leroy d'Etiolles on this subject, and which seem to prove that the lens was reproduced. Similar experiments since tried by Dr. Barkhausen, of Berlin, were, however, attended with different results. This subject has been still more recently investigated by M. Mayer, and the January number of the Archives Generales, contains a memoir from him in relation to it. M. Mayer examined the eye of an old woman, on whom the operation of couching had been performed several years previously. There was no trace of the depressed lens; the vitreous substance occupied its place, and immediately behind the anterior wall of the crystalline capsule, was observed the posterior wall or layer, with the vitreous humour pressing forwards upon it. The following experiments, among many others, were performed by M. Mayer. The lens was extracted from the left eye of a rabbit, which was killed three days afterwards. No trace of a new lens was found at this period, nor on the fourth, fifth, sixth, or seventh days; but on the eighth, the crystalline capsule contained a small ring of crystalline substance, which could be separated from the capsule. At the end of one month a large ring of crystalline substance occupied the place of the removed lens. In another rabbit, examined about the same time after the operation, a large white annular lens, with an opening in the centre, was found in the capsule, which adhered to this new lens. In eight weeks the new crystalline presented several white granular points arranged in a circle, having an opening in the middle; and in four months and a half it was not yet completely regenerated; for it was deficient at the centre, leaving there a rounded aperture, at the place where the capsule had been cut during the operation.

Soemmering has given us an account of four dissections at different periods after the operation on the human subject.

In the first, the patient had been couched eight years and a half before his death. In the place of the crystalline capsule, two semilunar whitish cheesy formations were formed, attached by their peripheral margin to the zonula Zinii, and floating free at the inner margin; they were doubtless the remains of the crystalline capsule. The new crystalline was transparent, gelatinous, and imperfectly formed. The former one had been completely absorbed, but a small piece of the original capsule was found imbedded in the vitreous humour.

Case II. *Three months after Couching.* In the place of the former lens Soemmering observed an annular transparent gelatinous deposit, imperfect at the centre, which was occupied with a fine, almost diaphanous and arachnoid membrane, situated right behind the pupil, and forming a septum between the aqueous and vitreous humours.

Case III. *Two years after Couching.* Similar appearances were discovered. A ring of transparent substance, of the consistence of jelly, in the situation of the lens of the left eye; in the right one, which had been also operated on, the new deposit was only semicircular, the upper part of the circle being deficient. Probably the cause of this was that, during the operation, the upper half of the capsule had been completely torn from its adhesions.

Case IV. *Three years after Couching.* The annular "renflement" or new deposit, had been very regularly formed; it was slightly and equally convex on both its surfaces, and was quite free from any adhesions to the uvea.

It is to be kept in mind that in order to display the annular crystalline substance the eye must be immersed in strong alcohol, by which the new deposit is rendered slightly opaque. Soemmering was at first puzzled to determine whether it was really a substitute for the removed lens, or was merely a product of inflammation, but he was speedily satisfied that the former was the case. Sometimes the ring is imperfectly formed; and in other cases we find only isolated points or grains. These cannot be the debris of the original cataractous lens, as some have imagined, for the simple reason that these grains are perfectly transparent, and the cataract was opaque. The preceding facts sufficiently show that there is a reproduction, although an imperfect one, of the crystalline lens; but we have reason to believe that an indispensable condition is a sound and healthy state of the capsule, and especially of its front layer; if this be either much torn and destroyed, or if it be rendered opaque by disease, there is no regeneration of the crystalline. In all probability, the secretion of the new substance is chiefly, if not altogether, from the inner surface of the anterior wall or layer of the capsule; and as this layer adheres intimately to the contained crystalline, no traces of the cavity or liquor of Morgagni can be henceforth discovered. The process of regeneration proceeds invariably from the circumference to the centre; and is always found interrupted at the place where the capsule has been cut or lacerated during the operation; the rent in the capsule is occupied with cellular substance. Hence the crystalline substance is never entirely reproduced, but always presents in the centre, or opposite to the injured part of its capsule, an opening which is filled up with a fine cellular tissue. The shape of a new crystalline is generally that of a three-quarter moon, the horns of which nearly touch each other. In the experiment on the rabbit, which was allowed to live for four months and a half after the operation of extraction, the new crystalline had this form, with a free space in the middle, occupied by a cellular web.

M. Le Roy d'Etiolle and Soemmering state that they have found the new crystalline free and unadherent to its capsule; the observations which M. Mayer has made do not coincide in this respect with theirs; it is a point left open for examination. It is worthy of remark that the mass of the new crystalline almost always exceeds that of the original; but that the entire eye very generally becomes somewhat shrunk and contracted for some time after the operation. This shrinking is found to extend even to the optic nerve, and that, too, beyond the decussation as far as the thalamus. It is conjectured, however, that in favorable cases the eye and its appendages may resume their original volume.—*Archiv.Generales.*

9. *On the Bearing of Epidemics upon Medical Statistics, and Political Economy.*—After a close investigation of the subject, M. Villerme arrives at the following conclusions. "That epidemics diminish both in frequency and intensity in those countries which from a state of barbarity or ignorancc, pass to that of civilization, and also in those which pass from a semi-civilized condition to one in which civilization is at its acme. That the lower classes of society being more frequently attacked, consequently more frequently fall victims to the epidemic, than the middling classes, or those in good circumstances. That in causing the disappearance of epidemics, and in diminishing their force and frequency, civilization has attended in a great many situations both the maximum and minimum epochs of life, but more particularly the former or maximum. That in a given number of paients of all ages, the mortality will be found to be much greater in the very young and very old; so that in this respect the laws of epidemic mortality correspond with those ordinarily observed, it results from this, that those epidemics which attack the two extremes of life, are cæteris paribus, the most fatal. That the vaccine has effected at least in the thickly populated country of France, nothing more than alter the period of death, but in those countries the inhabitants of which are widely separated from each other, and who possess the means of subsistence in greater profusion than is absolutely necessary, it undoubtedly increases the population. We should not suppose, however, that it cannot contribute even amongst us, in any way to produce this increase. In substituting, during a given period, one infant who arrives at the age of maturity, for two who although they consume, die before they are able to produce any increase, the vaccine favors production; and consequently indirectly favors by the employment of its products, or the means produced by its employment, an increase of population. But this effect is very trifling in comparison with that usually attributed to the vaccine. All those measures by which the diseases of infancy are warded off, operate in the same manner; they moreover in suppressing one of the causes of death, lend additional activity to others. That in civilized countries, the most fatal epidemics produce but a transient diminution in the population; the vacancies which they create being speedily filled up, both by marriages and by births, which are proportionably more numerous than ever, and also by the influx of strangers who are induced to come in, by the numerous vacancies produced in the different professions, trades, &c.— But if epidemics do not commonly diminish the population of those countries which they ravage, or at most only for a short period, they at least exert a manifest influence upon the population and its movements; an influence which varies according as the epidemic occurs annually, or at larger intervals. In the former case, that is where the epidemics are annual, as is the case along the banks of water-courses, or in the neighborhood of marshes, the renewal of the population is observed to be more rapid, and the average term of life is shorter; there being very few who arrive at adult or old age. The population does not diminish, and for the simple reason, that marriages take place at a very early age, and in a given time, the number of births greatly exceeds that in other countries. The same space, which in a healthy country would be occupied by the same individual during a period of forty years, will in one in which the causes operating against longevity are particularly rife, be occupied by two or three different persons. And in consequence of these fatal epidemics, the average term of life is reduced to twenty, and in some places even to thirteen years. But supposing the number of individuals to be the same in two different countries, yet their relative usefulness may be very different; in the one, we may have a poor, infirm, sickly population, immense numbers of whom perish previous to the performance of any productive act, and who resemble, if we may be allowed the comparison, a capital lost at sea. Whilst in the other, on the contrary, we have a robust, strong, vigorous class, who may in truth be termed the bone and sinew of the land, and who generally speaking live to an advanced age, or by labor obtain every necessary for themselves and families.

In the second case, or where an epidemic suddenly appears in any spot which has heretofore escaped its ravages, or even should it be characterized by unaccustomed violence in a country which has never been exempt from it, a very sensible diminution in the population will be observed, and immediately afterwards, cæteris paribus, an extraordinary increase in the number of births and marriages; such is the disposition to production at this period, that those unions which have remained uninterrupted, and from which no farther increase was anticipated, have again become fruitful. Finally, not only the yearly number of deaths is diminished, but likewise the proportional number, as if in truth mankind had become more tenacious of life, or less liable to the attacks of the "fell destroyer.' Hence arises the old saying, that 'all epidemics of a fatal character, are followed by seasons of great salubrity,' every thing, however, induces us to believe, that this fact exists only in appearance. We know, that the disease attacks generally speaking only weakly or sickly individuals, and that it consequently leaves a larger proportion of healthy, able-bodied persons, so that although it increases the relative space, it at the same time adds to the period of existence of the individuals remaining. Moreover, this latter change, whatever may be the cause, always exerts as is well known, a sensible influence upon the period of life, as well as upon the number of births."—*Archiv. Gen. Dec.* 1832. *Am. Jour. Med. Sciences.*

10. *New and singular variety of Hermaphrodism*—Being a paper read before the Royal Academy of Medicine, Paris, by M. Bouillaud. Abridged from the Journal Univ. and Hebdom.—In the course of the last year there was carried into la Pitie, a cholera patient of the name of Valmont, a *widower*, sixty-two years of age, by trade a hatter, and said to be given to dram-drinking. He was in the last stage of cholera when he entered the hospital, and he died on the following day.

I shall omit all that part of the post mortem examination which only related to the disease of which this person died; the monstrosity which presented itself to us (Dr. Donne was with me,) is what I particularly wish to notice.

As we had no suspicion about the sex of Valmont, who was treated as a male while in the wards, it was with no small surprise that, upon opening the abdominal cavity, we found in it a *well formed uterus*. We then noted the anormal state of the genital organs, and had those parts of them removed and put in alcohol, which we desired to examine subsequently more at leisure, for the cholera was then raging in all its fury.

M. Manec afterwards requested permission to examine the parts. This gentleman's profound anatomical attainments are well known, and to him I am indebted for the following description, as well as for the plate by which the organs are represented.

In the region of the external genital organs there is a penis of middling size, terminated by a well-formed glans, and prepuce, by which it is covered. The opening of the meatus urinarius is, however, not at the summit of the glans, but towards its lower portion. The bursæ are small, but very distinct, and their integuments brownish, wrinkled and supplied with hair, as in the natural state; they have a raphe dividing them symmetrically, and extending from the prepuce to the anus—the raphe itself being more firm and prominent than is usual in man. The bursæ contain no testicles, nor are there any vestiges of these organs discovered.

The mons veneris is more round and plump than it is commonly found in males, and it is covered with a moderate quantity of long hair, advancing along the penis, as if to conceal the latter. In the pelvis there are two ovaries similar in their form and structure to those of a girl of fifteen or sixteen years of age. [M. Bouillaud in a note here differs from M. Manec as to the structure of these supposed ovaries, M. B. considering them not as vesicular, but rather fibrous, or as if they consisted of a sort of intermediate tissue between that of the testicles and the ovaries.] Two fallopian tubes are seen, with their attachments, as in a well-formed woman. The uterus, which seems complete in every respect, holds its usual place between the bladder and the rectum, and opens into a species of vagina, to be described presently. The cavity of the uterus has the aborescent wrinkles which are observed in women who have had no children. The os tincæ projects into the vagina, as in the normal state. The vagina about two inches long and of a middling compass, presents the rugæ very conspicuously which are peculiar to virgins. Towards the neck of the bladder the canal contracts; and about the membranous portion of the urethra it is converted into a narrow duct, which from below upwards opens into the urethra by a little orifice about two millimetres in diameter, so that the urethra becomes in fact a continuation of the vagina. There is nothing remarkable about the urethra beyond the point of junction: it is exactly that of the male—having even at its origin the appendage of a regularly-formed *prostate*. There are also the *verumontanum*, and the prostatic follicles present, but no trace of openings to the ejaculatory ducts could be found. Beyond the prostate the urethra is destitute of external covering for eight or ten lines. Farther on, a spongy tissue with a bulbous enlargement, becomes connected with the canal, accompanying it to its extremity, where it is lost in forming the glans. All this spongy portion is attached to the corpora cavernosa, which, strong and well-marked as in the male, are strengthened in their root by a muscular apparatus as complete, and perhaps even more efficient than in the male. The bulbo cavernous muscles in particular, are very long and thick. Cowper's glands are present also, as in the male sex.

Like the testicles, the vesiculæ seminales, and vasa deferentia, are completely wanting. From the inguinal ring nothing more than a dense cellular cord—a rudiment of the round ligament—proceeds, accompanied by a nervous thread and an artery. The volume of this artery is considerable, and it communicates by large anastomoses with the superficialis perinæ, and the branches of the external pudics. The external female organs are altogether wanting.

With regard to other points connected with the general structure of this extraordinary individual, and perhaps scarcely less striking than those of the genitals, so well described by M. Manec, I may observe that the body of Valmont, very diminutive for a male, presents a degree of plumpness and rotundity which gives it much of the appearance of a female. The hands and feet are small, and more like those of a woman than of a man. The pelvis is shallow and wider than it would be in a well proportioned man. The face is furnished with a tolerably thick beard, yet its general aspect is effeminate, and, perhaps owing to its equivocal character, rather repulsive. There is an abundant supply of fat lining the pectoral and abdominal cavities. The mammary glands are much developed—too much, indeed, for a man, and yet too little for a well-formed woman; the nipples are of the size usual in healthy females.

Looking to the general conformation and volume of almost all the other parts, we should say that this individual maintains a sort of *juste-milieu* between man and woman. The heart, however, was nearly as robust as that of a man of middle size and strength.

From Valmont's own account given upon entering the hospital, it appears he was a widower. Thus an individual, with the essential organs of a female—though with the external resemblance of those of a male—had been placed in the condition of a husband! Having a womb, did he menstruate? or rather, did he exhibit the phenomenon of being affected with hæmaturia regularly once a month? But if, as our most famous physiologists have stated, the fact of being endowed with certain parts indicates the exercise of certain functions, why need we waste time in calculating what must have been the conduct of Valmont? We cannot help thinking, however, that this extraordinary being must have found himself curiously circumstanced. Did the passions of the male, or those of the female, predominate? Did they alternately stimulate him? or was he neutralized by the equal influence of each?

It has been said that, *propter uterum solum mulier est id quod est.* How does the maxim hold with respect to Valmont—a reputed man—and a married man?

In a practical point of view, it may be asked, how should we be able to say, from simple inspection of the exterior, that such a person as Valmont was really an hermaphrodite? Have we the means of forming a diagnosis? The problem is no doubt a difficult one, yet even in the absence of further facts, we might probably venture to affirm *a priori*, that where an individual, as in the case just cited, has a penis moderately (*mediocrement*) developed, without any trace of testicles either in the scrotum or inguinal regions, and where, considered in other respects, this individual would seem to hold a middle place between male and female—in such a case, I say, one might venture to affirm, that there are in the interior of the body some of those organs peculiar to the female, and constituting in so far an hermaphrodite more or less complete; and our decision would be greatly corroborated, if it could be ascertained that such an individual had, during a certain period of life, a monthly discharge of blood, or menstrual fluid from the urethra.

In conclusion, the facts contained in this paper will probably remove all doubt as to the existence of *real hermaphrodism*, however *incomplete*. It is now, we should say, no more than fair to admit that individuals of the human race may be found, who partake at once the male and female type in respect to their genital organs and, with reference to the other organs common to each sex, present a sort of *mezzo termine* between man and woman; a variety of hermaphrodism which does not seem to have sufficiently fixed the attention of teratologists. Those who deny the existence of all other varieties except that to which they have given the name of pseudo-hermaphrodism, will charge us, no doubt, with a credulity amounting to superstition, for having, even in Valmont's case, admitted the existence of a particular kind of true hermaphrodism: but such a charge will affect us little. We must believe that of which we are persuaded, even though it were a miracle: and, perhaps, of all superstitions, (if we may be allowed the term in speaking of natural science) that is the most worthless, not which believes what does not exist, but which refuses belief to what really does. To conclude in the language of a learned professor: "In matters like the present, which are at variance with received opinions, it is the part of wisdom not only to refuse acquiescence in what has not been rigorously proved, but not to fix too narrow limits to the powers of nature."*—*Lond. Medical Gazette, May*, 1833. *Balt. M. and S. Journal.*

11. *Extirpation of a degenerated ovarium*—By Dr. Erhardtsteim.—Agathe Duerr, a healthy peasant, aged 31 years, had been favorably delivered four times. Towards the conclusion of her fifth term of gestation, the abdomen became so much enlarged that it was supposed she carried twins. She was, however, only delivered of one female child, and the abdominal tumor merely subsided partially. Five days subsequent to this, Surgeon Ritter was consulted, who found the abdomen largely, but uniformly tumefied, and presenting evidences of fluctuation. In the left iliac fossa a hard tumor could be felt, but its character could not be determined on account of the swelling. Ritter supposed that there might be an extra uterine fœtus. Fourteen pounds of serosity were evacuated by paracentesis, after which he discovered an indurated mass into which he plunged the trocar, and drew off twelve pounds more of water. It was then ascertained that the tumor was formed by a diseased ovaria, and extended from the right ilium to within a small distance of the umbilicus; it presented a hard uneven surface. Ritter resolved upon extirpating this mass, which he did eighteen weeks after the accouchment. The skin was incised over the rectus muscle; this muscle was then divided, and its lower part detached from the peritoneum, and an incision made through that membrane large enough to admit the hand. Some difficulty was experienced in detaching the tumor from its surrounding attachments, but this was finally effected, and at the expiration of fifteen minutes it was withdrawn from the abdomen after having applied ligatures to three blood vessels. A twelve tailed bandage was immediately applied, sponges immersed in cold water were laid over the wound, and the extremities were enveloped in warm flannels. At the expiration of an hour the bandage was removed, and the wound had contracted one third, but a portion of the omentum was found protruding. The latter was reduced, the edges of the wound closed by sutures, and the bandage reapplied. On the day after the operation the patient was affected with repeated syncope, succeeded towards evening by rigors and a feeling of suffocation. It was discovered on the following day,

*M. Dupuytren, in a report on a human fœtus found in the mesentery of a boy of 14.

that contrary to the advice of her physicians, she had continued to nurse her child up to the period of the operation. It was now directed to be again applied to the breast, which occasioned a subsidence of the symptoms. But the secretion of milk ceased; a violent fever continued from the third to the eighth day, and she was affected with great oppression, extreme prostration, tension and tenderness of the abdomen, involuntary digections by stool, paucity of urine, facies Hippocratica, insatiable thirst, bilious vomiting, anxiety and delirium. An insurmountable disgust precluded the employment of internal medicines; fomentations of warm vinegar, however, which were renewed every two hours, provoked a free perspiration by which the patient was much relieved. A considerable quantity of bloody serosity was discharged from the wound on the eighth day, and she recovered her senses. The escape of a large quantity of gas was followed by a mitigation of all the symptoms. (Fomentations to the abdomen, enema.) The discharge from the wound continued on the ninth day, and ceased on the twentieth. A marked amelioration in the meantime took place, and the sutures were removed. On the eleventh, a lactescent discharge took place through the small openings left by the sutures which continued to the ninth week after the operation. During this time all the unpleasant symptoms subsided, and by the ninth week the wound was cicatrised. The tumor weighed twelve pounds. It was covered by a kind of lardacious membrane somewhat tendinous in its character, composed of several layers. The body of the tumor consisted of spherical portions of a lardacious consistence and of variable dimensions, some of which were excavated and filled with serosity. The orifices of the vessels which had been tied, were as large as a writing quill. On the side which corresponded to the ilium, two pouches were discovered, in which the serosity had been contained which was evacuated by the paracentesis.—*Arch. Gen.*—*Ib.*

12. *Case of hemorrhage of the uterus, arrested by compression of the descending aorta*—By Dr. Loewenhard, of Preslau.—Guided by theoretical views, Ploucquet was the first to advise compression of the descending aorta, in cases of hemorrhage of the uterus. Walter, James, (London Medical Repository and Review, 1825 and 1828,) and Ulsamen, insist upon the excellence of this method, and cite, in support of it, examples drawn from their own practice. In France, M. Baudelocque has contributed the most to make it known, in associating its use with that of spurred rye internally, in cases of hemorrhage produced by the separation of the placenta. The following case appears so conclusive, that we do not hesitate to offer it to our readers. The combination of many energetic means ought to be resorted to only in cases in which alarming floodings threaten to terminate the life in a few moments. A woman of thirty-two years, of a delicate complexion, was delivered on the sixth June, at twelve o'clock, of a fine child. Instead of the placenta, the midwife observed a stream of blood augmenting constantly in volume. She endeavored to detach the placenta and stop the hemorrhage; but in vain. At half past four, consequently five hours and a half after the commencement of the flow, the reporter was called. The patient resembled a corpse; the face was pale and cold, as well as the hands; the pulse scarcely perceptible; speech unintelligible; the blood flowed in so great abundance that the umbilical cord could not be seen hanging from the vagina. The hand was immediately introduced into the womb, and as the aorta beat forcibly, (a fatal sign in hemorrhage,) the reporter compressed strongly this artery against the vertebral column; the blood ceased instantly to flow; at the same time, the midwife threw injections into the vagina, of vinegar and water, and the patient took occasionally a spoonful of the following mixture:

Recipe. Water acidulated with hydrochloric acid. oz. j.
Tincture of catechu.
Tincture of digitalis, $\overline{a.a}$ oz. ij.

At the end of a quarter of an hour, it was attempted to detach the placenta, which proved a tedious process, because, at first, the blood flowed as soon as compression was removed; then, because the patient felt severe pains, and the uterus beginning to contrast, rendered the operation difficult. The separation was finally effected, and the hemorrhage successfully arrested. The patient so far recovered as to be able to nurse her child.—*Journal de Siebold.*

A similar case occurred in the hospital of St. Louis. After vain efforts to detach the placenta, the aorta was compressed externally above the umbilicus, and thirty grains of spurred rye were given to the patient. The cessation of the hemorrhage was not as prompt as in the preceding case; but, instead of flowing in a full current, the blood now formed a thread-like stream. The action of the spurred rye was felt at the end of twenty minutes after the placenta was expelled, and the hemorrhage ceased. It was time, for the patient was dying. She left the hospital at the end of fifteen days. It belongs to experience to decide between the two modes of compression; the one, external, through the abdominal walls; the other, internally, of the uterus, and through its posterior parietes. However, we may, a priori, and according to the anatomical relation of the parts, decide in favor of the first method. At the first trial, it may appear difficult to reach the aorta through the abdominal walls; this is, however, not the case. After the accouchment, the two anterior recti muscles of the abdomen are separated several inches; the muscles themselves are attenuated and spread out, so that the abdominal wall is formed along the whole length of the linea alba, only by the skin, the aponeurosis, and the peritoneum; the compression is made very exactly, and with the greatest facility. Whilst this compression is being executed, the vagina remains free, and we may, if desirable, cause the hand of an assistant to be introduced for the purpose of detaching the placenta, or to make injections, &c. Through the uterus, the operation is difficult, because the hand is compressed and cannot be placed in the most convenient position to act efficaciously. Instead of compressing the aorta perpendicularly, the hand is obliged to act under the disadvantage of a horizontal position, which is very fatiguing.—*Revue Med., Feb.* 1833. —*Ib.*

13. *Physiological Effects of various Gases upon the Animal System.*—In our fourth volume, p. 479, we noticed the experiments of Mr. Broughton on the physiological effects of oxygen gas upon the animal system. Mr. B. has since instituted some comparative experiments with various other gases; and the following are the results, as communicated to the British Association for the Advancement of Science, at their second meeting. In nitrous oxide, excitement followed by debility, bright redness of the interior of the body, long-continued action of the heart and intestinal canal after sensibility has ceased, and generally effects very similar to those produced in oxygen, took place in a much shorter space of time. Young rabbits are affected in little more than a minute, sparrows in four or five minutes; cold-blooded animals remain a long time unaffected, but ultimately die; a kitten left in the gas half an hour was past recovery.

With regard to other gases the author states his experience to be at variance with a prevailing notion that they are all incapable of entering the lungs, from a closing of the epiglottis simultaneously with the first-drawn inspiration. About thirty seconds of time are sufficient to manifest the effects of chlorine when the animal falls down insensible. If immediately opened, the heart is found palpitating, and the peristaltic movements going on. This gas is traced into the lungs both by their deep yellow tinge and acquired odour, and the brain likewise smells strongly of it.

Sulphureted hydrogen destroyed sensibility in about half a minute, and in two minutes and a half the heart still palpitated. The lungs and brain exhibited a dark brown tint, and smelt strongly of the gas.

In the other gases, animals do not remain unaffected so long as a minute, and contractility is not preserved, as in experiments with oxygen and nitrous oxide, although the period of its surviving sensibility and the motion of the diaphragm may vary a little. All the gases experimented on probably passed into the lungs, with the exception, perhaps, of the carbonic acid gas, immersion in which is borne without any very sensible effects during nearly three minutes, when the animal struggles, and falls down insensible, the blood appearing very dark-coloured, and the heart still and flaccid. From these results, the author extends his deduction of the poisonous character of oxygen in excess to the other gases which enter the lungs, and remarks on the specific analogy which obtains between the effects of nitrous oxide and fermented liquors.—*Second Report of the British Association for the Advancement of Science.—Am. Journ. Med. Sciences.*

13. *Professor Weber's Experiments on the Sensibility of the Skin.*—The Edinburgh Medical and Surgical Journal, for July last, contains an interesting account by Dr. Allen Thomson, of these experiments. It is a fact well known to physiologists, as Dr. Thomson observes, that there is a considerable difficulty in pointing out with certainty, when unaided by sight, any spot on the skin that has been touched, and in distinguishing how much of the common feelings of touch is due to the sensibility of the skin, and how much is derived from the muscular sensation produced by the motion of our limbs. It is also well ascertained that some parts of the skin are better adapted than others, either from their original structure, or in consequence of their being more exercised, to convey to the mind an exact impression of the physical qualities of the bodies with which they are brought in contact. It must be allowed, however, that our knowledge respecting this part of the physiology of the sense of touch is by no means definite.

Professor Weber of Leipsig has lately performed a very simple and ingenious set of experiments which illustrate the subject of the sense of touch, and furnish us with a mode of measuring with considerable accuracy, the relative acuteness of this sense in different parts of the skin of the same or of different individuals.

These experiments consist in placing the two points of a pair of compasses at different distances from one another, and in various directions, upon different parts of the skin of an individual who is not permitted to see the bodies touching him.* Professor Weber thus found, that, according to the distance of the two points from one another, we may have the feeling either of one only or of two tangent points, and that the distance at which we become sensible of the double impression as in the inverse proportion to the acuteness of the sense of touch in the skin; or, in other words, that we recognise a double impression made on very sensible parts of the skin, although the points are situated very near one another, while in those parts of the skin in which the sense of touch is obtuse, the points may be removed to a considerable distance from one another, and yet convey to us the feeling of only one impression.

Professor Weber has embodied the principal results of his experiments on the varieties in the acuteness of the sense of touch of different parts of the skin in eight propositions, of which the following is an abstract.

*Prop.* 1. The different parts of the skin or organ of touch do not possess an equal power of distinguishing two bodies by which they are touched at the same time. The distance of the two touching bodies being known, the degree of this power may be measured; for it is ascertained that if the organ of touch does not perceive the contact of two bodies when they are near one another, it becomes sensible to the impressions of both when the distance between them is increased.

If the touching points are sufficiently distant, we not only distinguish the impressions of both, but also the direction, longitudinal or transverse in relation to the body, in which they are applied to the skin. When they are brought nearer to one another they first give the sensation of the contact of a long body, but when brought still closer together, they appear as a single point upon the skin.

The ends of the fingers and the tip of the tongue have the power of distinguishing the distance of two points nearly equal, and in a much greater degree than any other part of the body. At two-fifths of a Paris line we are capable of distinguishing the longitudinal from the

*The sharp points of the common compasses may be blunted with a little sealing wax, which will have the effect also of taking away the cold feeling of the metal.

transverse position of the points on the tip of the tongue. At half a line two impressions are felt, more especially when the points are made to touch at the same time the upper and lower margins of the tongue, or the dorsal and palmar sides of the fingers; but in most other parts of the body this is different; for

*Prop.* 2. In many parts of our bodies we perceive the distance and situation of two points touching us at the same time more distinctly when they are placed parallel to the transverse than to the longitudinal direction of the body.

This may easily be tried in the middle of the arm or forearm: here the two points may be distinguished at a distance of two inches when placed in a direction across the arm, but they appear as one at this distance, or even (in some persons) at three inches when placed longitudinally.

*Prop.* 3. In those parts of our body in which the impressions of both points are clearly distinguished, although not distant, the space between these points appears to be greater than in other parts possessing a less sensible touch.

The experiments illustrative of this are very striking. They may be best performed by drawing both the points of the compasses gently along the skin, from a sensible to a less sensible part, or *vice versa;* as from the hand along the fingers, from the cheeks or ear across the lips, and towards the nose; from the jaw to the chin, from the occiput to the sacrum, with a point on each side of the median line, and from the chin to the pubis, in the same manner. In passing over the more acutely sensible parts, the points of the compasses seem to open or to recede from one another, and the reverse takes place in those regions in which the sensibility is obtuse.

*Prop.* 4. If the points are placed on two contiguous parts which may be moved voluntarily and independently of one another, the double impression is much more clearly perceived, and the points appear more remote from one another, than if at the same distance, they were brought in contact with one entire part. This is easily shown on the lips, fingers, and eyelids.

*Prop.* 5. We distinguish the two points more clearly, if they are brought into contact with two surfaces having a different structure and use, than when they are applied to one and the same surface.

This rule also holds in respect to surfaces possessing different degrees of sensibility; for in this case also, the points are more clearly distinguished when they touch two contiguous surfaces of different powers, than when they are both placed on the most sensible of them. This may be seen on the lips, by placing one point on the internal, and another on the external surface, in which position the points are distinguished at a smaller distance than in any other, although the surface of the lips directed towards the gums has a much less acute sense of touch than the red part. The same is the case with the white and red external surface of the lips.

To the same general rule may be referred another fact, viz., that a smaller distance of the points is perceptible when they touch at once the palmar and the dorsal surfaces of the fingers, than when they are both applied to one of these surfaces; and it may also be stated, under this head, that this power of distinguishing the points is generally greater when they are applied at equal distances on each side of a median line of the body.

*Prop.* 6. If we examine attentively the degree of acuteness of the touch in each part of the body, we shall find that this varies not only in the larger parts, but that there are also small spaces, in some of which the sense is more acute, in others in the immediate neighborhood, more obtuse. These points, however, do not vary to a great extent in the degree of their acuteness, nor has Professor Weber discovered any fixed order according to which they are disposed.

This observation would seem to show that the nervous fibriles are not quite equally distributed throughout the skin.

*Prop.* 7. If we are touched with greater force by one of the points than by the other, the impressions of both are distinguished less easily; for the stronger obscures the weaker.

*Prop.* 8. We distinguish two separate impressions more easily when they are not made exactly at the same time; and on this account, in performing all the experiments previously referred to, it is necessary to pay great attention in order to make the contact of both points synchronous.

The cause of the diversity in the sense of touch in different parts of the body is as yet unexplained. It is sufficiently obvious that the greater sensibility of some parts of the body does not depend on their being more frequently seen than others, as some have supposed to be the case; the middle of the back of the hand, consequently exposed to view, is surpassed by the fingers and palm, and even by the lower end of the fore-arm; the same is the case with the dorsum of the foot. The skin over the os sacrum and coccyx, though beyond the range of vision, is comparatively very sensible. The sensibility of the submental surpasses that of the sternal and abdominal regions; and, though the anterior is generally more sensible than the posterior surface of the body, this would appear to be connected with the structure of the skin rather than with the sight, for the sacrum and coccyx are more sensible than the pubis. Examples of blind persons also, and the great improvement their organs of touch are susceptible of from exercise, sufficiently show that sight has very little to do with our power of distinguishing by touch different regions of the kin. Nor does this power appear to depend chiefly on any mechanical advantage of one part over another, as, for example, that some parts are fixed on bones, and others very moveable. The tip of the tongue and free part of the lips which are loose, and the points of the fingers which are fixed, are possessed of nearly equally acute powers of touch.

The cause of these variations is probably to be sought for in the structure of the skin, with which subject we are as yet, as regards the distribution of the nerves at least very imperfectly acquainted. It seems to be obvious, however, that the great power of touch does not depend on the presence of papillæ, for the mammæ and some other parts with numerous papillæ have yet a very blunt sense of touch.

The tongue has papillæ over its whole upper surface; but it is only at the tip that the sense of touch is very acute.

Many experiments show that the direction of the course of the larger and smaller nervous twigs has some influence over the power of the skin, by which we distinguish the separate impressions of the points. The greater power which we have of distinguishing the points in a transverse than in a longitudinal position on the arms and legs, while on the face and some parts of the trunk of the body a position of the points parallel to the longitudinal direction of the body gives the clearest double impression, would seem to show that in general the feeling of the distance of the points is most acute, when they are applied across the direction of the nerves in their course. There are, however, other varieties which cannot be so easily explained in this manner, and it becomes necessary to have recourse to the supposition, that the quantity of nervous matter, as well as the mode of its distribution in the skin, may influence to a considerable extent the acuteness of the sense of touch. Sufficient attention has not as yet been given to this part of the subject.

The effect of motion of our organs, and of the bodies touching them, in augmenting the acuteness of the sensation, is very remarkable. When two points, for example, placed upon the skin appear as one, we can often recognise their double impression by moving the skin. It is thus that by moving the fingers we discover the asperities on surfaces which could not be felt, were the finger held at rest over them. We also acquire a more acute knowledge of the nature of an impression, by having it made on different parts of the skin in succession. By a peculiar internal feeling, called the muscular sensation, informing us of the extent of muscular contraction, we come to know the direction and space in which our limbs are moved; and every one knows that this feeling is of very considerable importance in aiding the sense of touch, and in improving that kind of touch frequently distinguished in this country by the term tact. It has already been remarked, that it is not unfrequently difficult to discriminate whether we judge of the qualities of a surface by the sensibility of the skin, or by the muscular sensation. We can in general tell immediately the direction in which any one pulls the hair of our head; but the knowledge of this direction is not derived, as might be supposed, from the sense of touch, but depends on an exertion of the muscles of the head, which is immediately and insensibly made with the view of resisting the motion of the head, which without it would occur. On fixing the whole head, it will be found that the power of distinguishing the direction still remains, though in a less degree. This seems to depend on the position of the skin in the neighborhood being altered by traction, for when we fix the skin the power of distinguishing the direction in which the hair is pulled entirely disappears.

Another illustration of this is obtained from the following experiment. Shut the eyes, hold the hand steady, and let some one touch your fingers with, and carry along their points various substances, as paper, glass, metal, wood, quill, leather, linen, silk, or velvet; you will be surprised how often you mistake the one for the other, according as they are more or less lightly pressed against the fingers. Metals when of the same temperature as the hand can scarcely be distinguished from glass and other substances with a smooth surface. When the finger of one person is conducted by another into a fluid, the slight pressure over a considerable surface informs him of its presence. If a person draws a plane surface along the finger of another, pressing at first gently, then gradually more strongly, and again gently, the feeling of a convex surface will be communicated to the finger, and that of a concave surface may be given by the greatest pressure being made at each end.

Professor Weber has also instituted some experiments for the purpose of ascertaining how far we are capable of judging of the weight of bodies by the sense of touch in the skin, and how far it is necessary that we should be assisted also by the muscular sensation; for it is obvious that in general we make use of both these means to obtain a correct estimate of weight. He found that when two equal weights* are placed on corresponding parts of the skin, he might add to or subtract from one of them a certain quantity without the person on whose skin they were laid being sensible of any change or inequality in them. He ascertained that when the hand or any other movable part of the body is laid quite inactive on a table, a much greater change can be made in the relative weight of the two bodies, without its being perceived, than when the limbs are free and capable of muscular exertion: that thirty-two ounces or drachms, for example, may be altered by from eight to twelve, when the hand is motionless and supported, but only by from one and a half to four, when the muscles are in action; and hence Professor Weber infers, that the measure of weight by the touch of the skin alone is more than doubled by the assistance of the muscular sensation.

By these experiments it was found that the lips estimate weight more correctly than any other part of the body: the fingers and toes may be reckoned next, the second phalanx being inferior to the third, and the first to the second: the palm of the hand and sole of the foot, especially the parts covering the ends of the metacarpal and metatarsal bones, possess also a considerable power, while the back, thorax, abdomen, scapulæ, arms, legs and occiput have very little power of estimating weight; which observations obviously show a considerable correspondence between those parts of the skin possessing the most acute sense of touch, and those estimating weights most correctly.—*Arch. Gen. from Rust's Mag.—Am. Journ. Med. Sciences.*

15. *Berzelius on the Chemical Constitution of Urine in Various Diseases.*—During the first period of fevers, the cutaneous transpiration being obstructed, the urine becomes more aqueous than in its healthy state: when the heat of the body increases, with acceleration

*The weights employed ought to be made of the same material, and must present the same size and form of surface to the skin. In order to insure this, and to correct the difference of temperature, it is well to interpose similarly shaped pieces of pasteboard between the weights and the skin.

of pulse, the urine becomes deeper colored, without, however, letting fall a deposit, while its acid reaction diminishes, and at last nearly or entirely disappears; it is then rendered turbid by the addition of bichloride of mercury, which does not happen when the acid is present. As the disease advances the urine becomes more saturated, and is then rendered turbid by a solution of alum. When albumen is secreted more copiously, it is troubled by nitric acid and heat. When the fever ceases, as, for instance, on the seventh day, the free acid suddenly reappears, the colour of the urine deepens, and it forms a deposit by cooling. This deposit is not an evacuation of morbific matter, but is merely a combination of red colouring matter, with uric acid or urate of ammonia, and perhaps nitric acid, in an unknown state of combination. In intermittent fever the urine presents these phenomena at each paroxysm, and then the deposit assumes a carmine tint. During slow nervous fever, there is constantly formed an abundant deposit of uric acid, containing little colouring matter; the urine then contains an excess of the phosphates and a deficiency of urea, the other ingredients being in their normal proportion.

In anasarca, which is generally the result of debility of the whole system, serum is effused into the urinary passages; hence the urine appears albuminous, and is troubled by bichloride of mercury, although much free acid may be present. After a short time, the kidneys appear to secrete an albuminous fluid, which occasions the urine to be precipitated by a solution of alum, nitric acid, or heat. As the albumen increases in quantity, the urea diminishes, and finally disappears altogether. These phenomona likewise appear in chronic hepatitis, dyspepsia, and towards the close of pulmonary affections, especially during the last stage of hectic fever.

During severe vomiting, whether from scirrhus of the stomach or other causes, the urine is frequently turbid, and has a milky aspect, letting fall a white deposit, which, when collected, appears mucilaginous, and by desiccation becoming first yellow and translucent, then white and pulverulent; by affusion of water it resumes its mucilaginous form; pure potass dissolves out of it mucus, leaving a residue of phosphate of lime. Hydrochloric acid dissolves the latter and renders the mucus transparent, which also dissolves by digestion. This state is generally accompanied with alkaline urine, arising from the presence of the carbonates of soda and ammonia, diminished quantity of uric acid, and an excess of urea. In gout the urine is usually very acid, except during the paroxysms, when it becomes alkaline or neutral; uric acid is always present in considerable excess; the deposit is also abundant by cooling.

In jaundice the urine appears yellow, from an admixture of biliary matter; and on the addition of nitric acid, a play of colours is generally produced. Hydrochloric acid renders it green or brown, according to the state of modification in which the biliary matter exists.

Sometimes orange-yellow flocculi are deposited: these are soluble in caustic potass, and give the usual reaction with nitric acid, in partial hepatic obstructions, when no discoloration of the skin has appeared: the bile, by passing through the lymphatic vessels of the engorged parts, enters the circulation, and may be detected in the urine by evaporating a portion of the latter, digesting the extract in alcohol of specific gravity .833, and letting the tincture evaporate to dryness. The addition of nitric acid will then produce the change of colours—viz. green, blue, violet and yellow—which characterizes the colouring matter of bile. In spasmodic and hysteric affections, the urine often becomes limpid and colourless, being, indeed, merely a solution of the urinary salts, deprived of almost every particle of organic product.

In diabetes mellitus, an immense quantity of sugar is secreted. At the first access of the disease, the only symptoms are copious emission of urine and diminished appetite; the cutaneous transpiration is obstructed, and the urine is supplied with water from all the fluid ingesta. The specific gravity is often as high as 1.050; as the sugar increases the urea diminishes, and at last totally disappears: colour pale yellow, taste sweet, odour like that of skim-milk; the inorganic salts are present in their natural proportion, being merely diluted with a larger quantity of fluid. Towards the close of the disease, when hectic fever makes its appearance, the urine becomes albuminous, and now passes spontaneously into the alcoholic fermentation; the quantity of urine expelled increases to an enormous amount. We may calculate by the following formula the weight of the dry extract in solution in the urine, and of course the quantity daily expelled. A pint of urine, specific gravity 1.020, leaves by evaporation 382.4 grains of dry extract; which weight increases in the proportion of 19.2 grains for each unit of specific gravity, until the latter amounts to 1.050; so that if we have a patient voiding urine of specific gravity 1.021, we can determine the weight of solid matter present in his urine by making this calculation, 382.4+19.2=401.6 grs. in every pint. When diabetic urine is evaporated to dryness, and alcohol digested on the residue, the sugar and extractiform matter are dissolved; this solution, by repose, leaves either granular crystals, like grape sugar, or merely a honey-like mass. We are ignorant whether this difference is owing to a diversity of saccharine matter, or to the presence of a deliquescent matter, which prevents its crystallizing. Sugar has been sought after in vain in the blood of diabetic patients: indeed this disease appears to be seated solely in the kidneys, which convert almost every portion of organic matter arriving in those viscera into sugar. When a favorable change takes place, urea reappears, and a quantity of albumen makes its appearance in the urine.

M. Meisner, who has paid great attention to this subject, gives the following quantitative analysis of three specimens of diabetic urine, taken from the same patient at different periods:—

| | 1. | 2. | 3. |
|---|---|---|---|
| Matter soluble in ether, urea lactic acid, lactate lime, extractiform matter - | 0.34 | 0.33 | 0.65 |
| Matter soluble in alcohol, diabetic sugar, extractive matter and salts - - - - | 7.06 | 3.46 | 5.78 |
| Matter soluble in water; extractive matter and salts - - - - - - - - - - | 1.37 | 3.44 | 0.99 |
| Vesical mucus, subphosphate of lime, and traces of peroxide of iron - - - | 0.34 | 0.31 | 0.46 |
| Water - - - - - - - - - - - - - - - | 91.19 | 92.46 | 92.10 |

In diabetes insipidus no sugar is found, but there is present in the urine a matter similar to that obtained by digesting alcohol on an aqueous extract of muscular fibre: no ultimate analysis of this matter has been as yet made.—*Lond. Med. Gaz.*—*Ib.*

16. *Observations on Local Blood-Letting, and on some New Methods of practising it*—By Jonathan Osborne, M. D., &c. &c.—Opening the veins of a foot is a practice still resorted to in cases of obstructed menstruation by practitioners who must be above the influence of vulgar prejudice on the subject. The trials which I have made have not enabled me to arrive at a conclusion as to the question whether this practice possesses any advantage above general blood-letting. Bleeding from the veins of the tongue is another old practice now nearly forgotten, having been superseded by the more manageable mode of taking blood by leeches. By opening the veins on the back of the hands we can usually obtain blood with great facility when particular circumstances forbid bleeding in the arm. Bleeding from the jugular vein is not well suited for taking blood from the head, because the external jugular, which alone is within our reach, is supplied from the superficial veins of the neck, and principally from those of the larynx, but not from the interior of the head. Great benfit, however, may be derived from opening it in sudden attacks of croup.

The application of leeches is frequently a cause of great fatigue to the patient, from the length of time during which stuping with hot water is kept up in order to promote the hæmorrhage from the leech bites; and, in some cases, when this operation is continued under the bed-clothes, the damp communicated to these produces cold, and is uncomfortable to that degree as often to prohibit their use. All this is obviated by the application of warm cloths of linen or calico applied perfectly dry, and removed in succession according as they have become saturated. By these means the blood is absorbed by capillary attraction, a process which cannot take place with wet applications. When dry cloths are thus applied and renewed to cuts in the skin, or to leech bites, I have found the bleeding uniformly to continue as long as the application was kept up, it being required only to supply fresh portions of the dry cloth to insure the continuance of capillary attraction, and thus to prevent coagulation at the mouths of the vessels.

This mode of managing leeches I am thus particular in describing, as it has enabled me to apply them in a case in which if wet cloths were used, very serious danger might arise. I allude to bronchitis, both acute and chronic, in which the application of leeches to the larynx and to the trachea in the triangular space between the mastoid muscles, has appeared to me to be the most decisive and immediately successful remedy of all those which I have ever employed. In laryngitis, their utility is obvious and commonly recognized, but in bronchitis it has escaped notice that the most immediate depletory process which can be performed on the mucous membrane on the bronchial tubes is that of leeching the trachea and larynx. It appears to remove blood not only from the mucous membrane of that part of the bronchial tube to which the application is made, but also from the whole tract of the bronchial tubes throughout their ramifications, being nearly equally efficacious in putting an end to the cough, when the remoter tubes are affected, as when the larynx is the chief seat of disease. This application is also of singular efficacy in stopping the cough of phthisis, insomuch, that by resorting to it according as required in cases in the hospital, we have been enabled to secure sleep at night, and during the day to keep the phthisical patients so free from cough, that a superficial observer might readily believe that we had cured the disease.

It has been ascertained that leeches will continue to live and to draw blood although immersed in water at a temperature considerably above 100 deg. Now, in cases of violent inflammation of the abdominal viscera, when local abstraction of blood and warm fomentations are both at the same time imperatively demanded, as soon as leeches have been applied to the abdomen the patient may immediately be placed in a hip bath without waiting for them to fall off. Thus we may cause the relaxation and diminution of sensibility produced by the heat to combine with the benefit to be derived from the topical loss of blood.

The application of leeches to mucous surfaces was, I believe, first described by the surgeon-general, Mr. Crampton. Although I have not met with any case of cynanche which required the direct application of leeches as advised by him, yet there can be no doubt as to the immediate benefit to be derived from it. I have resorted to the mode of applying leeches to other mucous membranes by passing a needle and thread through their tails, at about one-fourth of an inch from the extremity. This practice, so far from incapacitating them from action, causes them to bite with increased ardor, and, in fact, may be used to stimulate torpid leeches. The thread to be passed through the tail of the leech should be strong, and its extremities are to be held by the operator, while, if necessary, he may direct the mouth of the leech by a probe or channel made with a card, to the place where its services are required.

In certain headaches confined to the frontal sinus, which, although originally derived from derangements of the digestive organs, yet do not cease when those derangements have been removed, a prompt relief is obtained from applying leeches in this manner to the interior of the nostrils; and in those cases no benefit is usually derived from leeches externally applied. The bleeding is usually rather more copious than if the application had been made on the skin; if, however, it should be deficient, the patient may encourage it by breathing over the vapor of hot water.

In inflammations of the conjunctiva, a leech thus applied to the Schneiderian membrane of the adjacent nostril evidently unloads the vessels of the eye. This application I have found of great use after the previous application of leeches to the tarsal conjunctiva. It appeared to render the improvement derived from the latter permanent, and prevented the necessity of repeating it.

In inflammations of the ear, this mode of applying a leech inside the meatus is eminently useful; and next to it in importance is the application of them behind the ear as near as may be to the meatus. It may be objected, that such applications are not well suited to inflammations of the internal parts of the ear, inasmuch as those are supplied by a different set of vessels from the external. But the effect of leeches is independent of vascular connection. For example:—in inflammations of the stomach or intestinal canal, the benefit derived from leeches applied to the corresponding region of the abdomen is acknowledged by all; but the vascular connection between those parts is as remote as that between distant regions of the body, the one being supplied from the arteries arising from the abdominal aorta, and the other from the epigastric and mammary arteries; and that there can be no anastomosis of vessels is evident from the interposition of the peritoneum, which insulates the viscera completely from the anterior parietes of the abdomen. The same observation applies with the same force to the thoracic viscera and to the brain. In all those cases, however, the effect of local bleedings is proved so repeatedly in our daily experience, that the inability of satisfactorily explaining the way in which the effect is produced must not be allowed for one moment to press against the evidence of facts.

In inflammation of the mucous membranes of the bowels, especially of the rectum, the French practitioners apply leeches to the margin of the anus. If the leeches take externally, no benefit is derived; and to apply them internally is often difficult, on account of the violent contractions of the sphincter. Those contractions also prevent any considerable quantity of blood being obtained from the bites. I have employed a method of taking blood from the rectum which obviates these inconveniences.—*Dublin Journ.*—*Boston Journal.*

17. *On the Oil of the Croton Tiglium as a Purgative for Children.* —By Edward Augustus Cory.—It is a matter of the greatest importance, in the treatment of the diseases of children, that the remedial agents employed should be palatable to the patient. A disease is frequently aggravated considerably by the forcible administration of nauseous medicine, especially where the head and chest are affected; indeed, this remark will apply to the generality of inflammatory affections. It is well known, that the active principle of the cathartics, usually administered to children, is calomel, it being the least unpleasant to the taste; but this remedy I am convinced, from multiplied experience, does not completely answer the required end, unless it be given in combination with other aperients, as jalap, rhubarb, &c. &c., which render it extremely disagreeable to the little patient. One of the most pleasant and efficient purgatives for children, with which I am acquainted, is the ol. croton., prescribed according to the following formula:—

R. Olei crotonis, gtt. ij.

Sacch. albi, ʒ ij.

Pulv. acaciæ, ʒ ss.

Tinct. card. co., ʒ ss.

Aq. q. s. ft. mist., ℥ ss.

Of this a child, five or six years old, may take two or three teaspoonsful every three or four hours, until the bowels have been freely acted upon. I have been for some time in the daily habit of using this preparation in the treatment of the diseases of children, where a complete and speedy evacuation of the bowels is required. I have found it of admirable service as a purgative in cephalic and thoracic affections; it acts with great celerity and occasionally produces a gentle vomiting, which is often salutary. I do not recollect one single instance where its action has been violent and dangerous, when given according to the formula directed. I strongly recommend its general use, and I feel confident that it will become a favorite medicine in all the morbid affections of children, where a quick, certain, active, and pleasant purgative is indicated. It may be proper to remark, that the croton oil I prescribe is procured from, and, I believe, prepared by, Messrs. Drew, Heyward, and Baiss, wholesale druggists, Great Trinity lane, Bread street. It appears to be of excellent quality.—*Lond. Med. and Surg. Journ.—Ib.*

18. *Indian Ophthalmia treated with much success with Alum.*—M. Sonty, in a report which he lately made to the Minister of the French Marine, mentions his great success in the treatment of a most violent and rapidly destructive epidemic—purulent ophthalmia, in the East Indies. At first he had employed antiphlogistic measures, but they entirely failed, or rather the disease was too intense to be quickly enough affected by them. The natives employed very stimulating applications; as, for example, a mixture of pepper, lemon-juice, and the juice of tamarind leaves, to which is added afterwards, roasted walnuts; this paste they applied round the eyelids. M. Sonty soon found out the marvellously good effects of rock alum. He took a piece, with which he kept stirring, for eight or ten minutes, the white of an egg, which is then to be put into a white muslin bag. When this is to be used the patient's head must be held back, and while the eyelids are kept open, a few drops of the liquid are to be squeezed from the bag upon the eye. This operation must be repeated very frequently—in some cases every half hour. The same treatment is applicable in all the stages of the disease, and generally cures it in from 24 to 48 hours.—*Archives Generales.—Ib.*

